# Supplementary material for: The Systems Biology Research Tool: evolvable open-source software
Source: BMC Syst Biol. 2008 Jun 29;2:55. doi: 10.1186/1752-0509-2-55 (PMC2446383; doi:10.1186/1752-0509-2-55)
Supplement: Additional file 1 — SBRT Archive. An archive of the current version of the Systems Biology Research Tool. [file 1752-0509-2-55-S1.zip › sbrt-1.4.0/doc/developers_guide/api/sbrt/kernel/math/geom/package-use.html]

Uses of Package sbrt.kernel.math.geom


|  |  |  |
| --- | --- | --- |
| **Overview** \| **Package** \| Class \| **Use** \| **Tree** \| **Deprecated** \| **Index** \| **Help** | |  |
| PREV   NEXT | **FRAMES**    **NO FRAMES**     **All Classes** |  |


---


## **Uses of Package sbrt.kernel.math.geom**

| Packages that use sbrt.kernel.math.geom | |
| --- | --- |
| **sbrt.kernel.math.geom** | Provides classes and interfaces for creating objects relevant to computational geometry. |
| **sbrt.kernel.math.geom.comp** | Provides classes and interfaces for performing geometry-related computations. |
| **sbrt.kernel.math.geom.proc** | Provides classes and interfaces for performing geometry-related processes. |
| **sbrt.shell.mng.fba** | Provides classes and interfaces for creating Flux Balance Analysis process managers. |
| **sbrt.shell.mng.geom** | Provides classes and for creating geometry process managers. |

Classes in sbrt.kernel.math.geom used by sbrt.kernel.math.geom

****ConvexPolytope****
  
          This interface is used to represent convex polytopes that are represented
as the intersection of a finite set of half-spaces.

****HashConvexPolytope****
  
          This class is a concrete implemenation of `ConvexPolytope`.

****ParallelHyperplanes****
  
          This class is used to represent a pair of bounding parallel hyperplanes.

Classes in sbrt.kernel.math.geom used by sbrt.kernel.math.geom.comp

****ConvexPolytope****
  
          This interface is used to represent convex polytopes that are represented
as the intersection of a finite set of half-spaces.

Classes in sbrt.kernel.math.geom used by sbrt.kernel.math.geom.proc

****ConvexPolytope****
  
          This interface is used to represent convex polytopes that are represented
as the intersection of a finite set of half-spaces.

Classes in sbrt.kernel.math.geom used by sbrt.shell.mng.fba

****DimensionException****
  
          This class is used to indicate invalid dimensions.

Classes in sbrt.kernel.math.geom used by sbrt.shell.mng.geom

****ParallelHyperplanes****
  
          This class is used to represent a pair of bounding parallel hyperplanes.

---


|  |  |  |
| --- | --- | --- |
| **Overview** \| **Package** \| Class \| **Use** \| **Tree** \| **Deprecated** \| **Index** \| **Help** | |  |
| PREV   NEXT | **FRAMES**    **NO FRAMES**     **All Classes** |  |


---
